# Quantum Synchronization and Entanglement of Dissipative Qubits Coupled to a Resonator

**DOI:** 10.3390/e26050415

**Published:** 2024-05-11

**Authors:** Alexei D. Chepelianskii, Dima L. Shepelyansky

**Affiliations:** 1 LPS, Université Paris-Sud, CNRS, UMR 8502, 91405 Orsay, France; 2 Laboratoire de Physique Théorique, IRSAMC, Université de Toulouse, CNRS, UPS, 31062 Toulouse, France

**Keywords:** quantum synchronization, qubits, entanglement, quantum dissipation, Lindblad equation, Jaynes–Cummings model, driven resonator

## Abstract

In a dissipative regime, we study the properties of several qubits coupled to a driven resonator in the framework of a Jaynes–Cummings model. The time evolution and the steady state of the system are numerically analyzed within the Lindblad master equation, with up to several million components. Two semi-analytical approaches, at weak and strong (semiclassical) dissipations, are developed to describe the steady state of this system and determine its validity by comparing it with the Lindblad equation results. We show that the synchronization of several qubits with the driving phase can be obtained due to their coupling to the resonator. We establish the existence of two different qubit synchronization regimes: In the first one, the semiclassical approach describes well the dynamics of qubits and, thus, their quantum features and entanglement are suppressed by dissipation and the synchronization is essentially classical. In the second one, the entangled steady state of a pair of qubits remains synchronized in the presence of dissipation and decoherence, corresponding to the regime non-existent in classical synchronization.

## 1. Introduction

The synchronization of two maritime pendulum clocks, discovered by Christian Huygens in 1665 [1], forms the foundation for synchronization phenomena that appear in various systems ranging from clocks to fireflies, cardiac pacemakers, lasers, and Josephson junction (JJ) arrays (see historical survey and overview in [2,3,4]).

With the modern development of quantum computation and quantum information (see, e.g., [5]), the exploration of quantum synchronization phenomena in the quantum realm has become significantly important. The phenomenon of quantum synchronization gains even more relevance for JJ arrays and superconducting qubits where dissipative and quantum effects are important (see, e.g., [4,6]). A strong coupling regime involving several superconducting qubits with a microwave resonator has been experimentally realized [7,8,9,10,11], which requires investigating the effects of strong interactions in quantum dissipative-driven systems.

The essential element of synchronization in classical mechanics is dissipation, which leads to the phase synchrony of coupled autonomous systems with energy supply [2]. Thus, the analysis of quantum synchronization requires the use of formalism of dissipative quantum mechanics. This formalism is known and based on the Lindblad master equation for the density matrix ρ of the whole system developed in [12,13] and reviewed in [14]. It has fundamental grounds and allows us to study complex phenomena, such as quantum strange attractors [15,16]. However, its numerical simulation requires integrating time propagation of the whole density matrix with a large number of N×N components that are numerically rather heavy.

Another approach to dissipative quantum evolution is based on the method of quantum trajectories (see, e.g., [17,18,19]), which stores only a stochastically evolving state vector of size *N*. The averaging over several realizations allows us to characterize the probabilities from the density matrix with statistical fluctuations. The application of quantum trajectories to the problem of quantum synchronization has been reported in [20], where it was shown that—at small dimensionless values of the Planck constant *ℏ*—the synchronization remains robust with respect to quantum fluctuations. These model studies of quantum synchronization [20] were shown to have close similarities with Shapiro’s steps in the Josephson junction [4]. The quantum trajectory method has also been applied to investigations of quantum synchronization of one and two qubits coupled to a driven dissipative resonator [21,22]. It has been shown that the driving phase can impose quantum synchronization of qubit phases and mutual synchronization and entanglement of two qubits. Regarding the synchronization of classical systems, this corresponds to the regime of the phase synchronization of oscillators by external driving [23]. Thus, the phases of oscillators rigidly follow the phase of driving a monochromatic field. In a similar way, we define a quantum synchronization of qubits as the regime in which a phase (or orientation angle) of each qubit follows the phase of an external monochromatic driving field applied to a resonator (cavity).

The description of quantum trajectories involves significant quantum noise and fluctuations, making it difficult to obtain complete information about the evolution of the density matrix and its steady state using this approach. Therefore, it is crucial to study the phenomenon of quantum synchronization of qubits using the Lindblad master equation for the density matrix. In this work, we describe the results obtained with the Lindblad description.

In recent years, there has been growing interest in various aspects of the quantum synchronization of various systems (see, e.g., [24,25,26,27,28,29,30]). There are even discussions about quantum synchronization for satellite networks [31,32,33]. These developments initiated our research on the quantum synchronization of several superconducting qubits coupled to a driven dissipative resonator performed in the frame of the Lindblad master equation. There are also works on the quantum synchronization of oscillators (see, e.g., [34]), but here, we only discuss the synchronization of qubits.

They conducted extensive numerical simulations of tens of thousands of Lindblad equation components, enabling the establishment of various nontrivial regimes of quantum synchronization and entanglements involving one, two, three, or four qubits strongly coupled with a driven resonator in the presence of dissipation. It is shown that in regimes of either weak or strong dissipation, the system behavior can be well described by semi-analytical methods of weak perturbation theory or semiclassical theory, respectively. However, certain nontrivial dissipative regimes with preserved entanglement of dissipative qubits remain inaccessible for such semi-analytical descriptions.

At the time when Christian Huygens discovered clock synchronization in 1665, improving clock accuracy was a strategic military task for ship navigation, which saw significant enhancements based on this discovery (see [1,2,3])). We believe that quantum synchronization of qubits will also play a fundamental role in quantum computing. Indeed, with a decrease in size scales, the quantum and dissipative effects start to play more important roles (see, e.g., [4,6]). In such a regime, a quantum synchronization of several qubits becomes important for qubit control and the operation of quantum gates. At the same time, it is imperative to understand to what extent the dynamics of dissipative qubits become classical and, thus, it can be described in the semiclassical approximation, meaning that dissipation suppresses quantum effects, or that quantum features and entanglement resist and remain present even for dissipative qubits. Our results are obtained in the frame of the Lindblad equation for a driven dissipative resonator coupled with dissipative qubits, establishing the existence of two regimes: one when the synchronization of qubits is well described by the semiclassical approach so that quantum features are suppressed by dissipation and another when entanglement is preserved for dissipative synchronized qubits, thus being essentially quantum.

Our results are based on an extensive numerical and semi-analytical analysis of the Lindblad equation of the system of driven dissipative resonators coupled to up to four dissipative qubits. A certain advantage of the Lindblad description is that it allows obtaining the steady state of the system corresponding to infinite times while the approach of quantum trajectories always gives characteristics fluctuating with time and very long times are needed to obtain steady-state properties. This was the case of results presented in [21,22] with one or two non-dissipative qubits; this is not the case in experiments with superconducting qubits (see, e.g., [6,7,8,9,10,11]). Our results clearly show the presence of a synchronized steady state with up to four qubits. They also show that the entanglement of qubits can survive in the system’s steady state. We begin this article with the model description, followed by the introduction of semi-analytical methods, which allow for a better understanding of the physical effects appearing in the direct numerical simulations of the Lindblad equation of the system.

This article is organized as follows: Section 2 describes the model, numerical methods, and semi-analytical approximations. Section 3 presents the obtained numerical results and RWA validity tests of RWA; the results and validity of the weak damping rate equation approach are presented in Section 4 and those of the strong damping semiclassical regime are presented in Section 5. The regimes beyond the validity of semi-analytical approaches are analyzed in Section 6, the synchronization of several qubits is described in Section 7, and a discussion is presented in Section 8.

## 2. Model Description

We consider a model of several qubits (spin half systems) interacting with one harmonic cavity driven by an external monochromatic field. The Hamiltonian of this system is as follows:

(1) $$\begin{array}{cc} \hat{H}\left(t\right) & =\hbar \omega_{0}{\hat{a}}^{+}\hat{a}+\hbar \sum_{l}\lambda_{l}{\hat{\sigma }}_{x,l}\left(\hat{a}+{\hat{a}}^{+}\right)+\sum_{l}\frac{\hbar \Omega_{l}}{2}{\hat{\sigma }}_{z,l}+2F\left(\hat{a}+{\hat{a}}^{+}\right)\cos\omega t \end{array}$$

where $\omega_{0}$ is the frequency of the cavity, ω is the frequency of the driving field, and *F* is the driving strength. The operators ${\hat{a}}^{+}$ and $\hat{a}$ are the rising/lowering operators for the cavity photons. The qubits in this model are indexed by *l* (we consider various numbers of qubits, from 1 to 4); their contributions to the Hamiltonian are given by the Pauli matrix operators, where $\Omega_{l}$ is the energy splitting (Zeeman splitting for spin systems) and $\lambda_{l}$ denotes their coupling strengths with the cavity.

For F=0 and one qubit, the Hamiltonian (1) is reduced to the Jaynes–Cummings model [35], appearing in many systems of quantum optics and other physical systems [36,37]. It was experimentally realized with Rydberg atoms in a resonance cavity [38]. The unitary behavior of the Jaynes–Cummings model with a monochromatic-driven cavity was studied in [39]. However, here, we consider the model with one or several qubits in the presence of driving and dissipation, which corresponds to a real situation of superconducting qubits [6].

In the rotating wave approximation (RWA), the Hamiltonian in Equation (1) is as follows:

(2) $$\begin{array}{cc} \hat{H}\left(t\right) & =\hbar \omega_{0}{\hat{a}}^{+}\hat{a}+\hbar \sum_{l}\lambda_{l}\left(\hat{a}{\hat{\sigma }}_{l}^{+}+{\hat{a}}^{+}{\hat{\sigma }}_{l}^{-}\right)+\sum_{l}\frac{\hbar \Omega_{l}}{2}{\hat{\sigma }}_{z,l}+F\left(\hat{a}e^{i\omega t}+{\hat{a}}^{+}e^{-i\omega t}\right) \end{array}$$

where we kept only the resonant terms in both the driving field and spin-cavity interactions. The Pauli matrices ${\hat{\sigma }}_{l}^{+}$ and ${\hat{\sigma }}_{l}^{-}$ are the rising/lowering spin operators for spin/qubit *l*. For F=0 and one qubit, the Hamiltonian (2) is reduced to the RWA form of the Jaynes–Cummings model [35].

An advantage of the RWA is that the time-dependent Hamiltonian in Equation (2) can be made stationary by moving to the rotating frame using the unitary transformation, as follows: $\hat{U}\left(t\right)=\exp\left(i\omega {\hat{a}}^{+}\hat{a}t+i\omega \sum_{l}{\hat{\sigma }}_{l}^{+}{\hat{\sigma }}_{l}^{-}t\right)$ which acts on the density matrix $\hat{\rho }$, moving it into the rotating frame through ${\hat{\rho }}_{R}=\hat{U}\left(t\right)\hat{\rho }{\hat{U}}^{+}\left(t\right)$. This leads to a stationary rotating frame Hamiltonian [36,37]:

(3) $$\begin{array}{cc} {\hat{H}}_{R} & =\hbar \left(\omega_{0}-\omega \right){\hat{a}}^{+}\hat{a}+\hbar \lambda \sum_{l}\left(\hat{a}{\hat{\sigma }}_{l}^{+}+{\hat{a}}^{+}{\hat{\sigma }}_{l}^{-}\right)+\sum_{l}\frac{\hbar (\Omega_{l}-\omega )}{2}{\hat{\sigma }}_{l,z}+F\left(\hat{a}+{\hat{a}}^{+}\right) \end{array}$$

for simplicity, we assume that the coupling strength between each qubit and cavity is the same for all qubits; thus, $\lambda_{l}=\lambda$ for all *l*.

We describe the dissipative effects in the framework of the time-dependent Lindblad equation [14] with zero temperature dissipative terms for both qubits and cavity dynamics, as follows:

(4) $$\begin{array}{cc} {𝜕}_{t}\hat{\rho } & =-\frac{i}{\hbar }\left[\hat{H},\hat{\rho }\right]+L_{d}\left(\rho \right) \end{array}$$

where $L_{d}$ gives the dissipative part of the Lindblad dynamics:

(5) $$\begin{array}{cc} L_{d}\left(\hat{\rho }\right) & =\gamma \left(\hat{a}\hat{\rho }{\hat{a}}^{+}-\frac{1}{2}{\hat{a}}^{+}\hat{a}\hat{\rho }-\frac{1}{2}\hat{\rho }{\hat{a}}^{+}\hat{a}\right)+\gamma_{s}\sum_{l}\left({\hat{\sigma }}_{l}^{-}\hat{\rho }{\hat{\sigma }}_{l}^{+}-\frac{1}{2}{\hat{\sigma }}_{l}^{+}{\hat{\sigma }}_{l}^{-}\hat{\rho }-\frac{1}{2}\hat{\rho }{\hat{\sigma }}_{l}^{+}{\hat{\sigma }}_{l}^{-}\right) \end{array}$$

with γ being the dissipation rate of the cavity and all qubits have the same dissipation rate $\gamma_{s}$.

In the RWA formulation, Lindblad Equation (4) takes the following form:

(6) $$\begin{array}{cc} {𝜕}_{t}{\hat{\rho }}_{R} & =-\frac{i}{\hbar }\left[{\hat{H}}_{R},{\hat{\rho }}_{R}\right]+L_{d}\left(\rho_{R}\right) \end{array}$$

Since the Hamiltonian ${\hat{H}}_{R}$ is stationary, the steady-state value of ${\hat{\rho }}_{R}$ can be found by setting the left-hand side of Equation (6) to zero. The advantage is that finding the steady state does not require numerically integrating the equations of motion Equation (4) and can be found more directly by solving the matrix equation, as follows:

(7) $$\begin{array}{c} -\frac{i}{\hbar }\left[{\hat{H}}_{R},{\hat{\rho }}_{R}\right]+L_{d}\left(\rho_{R}\right)=0 \end{array}$$

The described model is an extension of the Jaynes–Cummings model, which appears in various fields of physics describing the most natural coupling between the oscillator and two-level atom (spin-half). The extensions we use include the dissipation of qubits and cavities and also the monochromatic driving of cavities. Such a situation naturally appears for superconducting qubits coupled to a driven cavity (see [6,7,8,9,10,11]) and, thus, we believe that our model is suited for the study of the synchronization of dissipative qubits coupled to a driven resonator (cavity).

### 2.1. Numerical Methods

For convenience, in our study, we use units where ℏ=1 and use the cavity–qubit coupling strength λ as the energy scale in dimensionless quantities. This choice of units is convenient for analyzing RWA validity because only relative energies to the excitation frequency appear in Equation (3) and, thus, energy differences rather than the absolute energy become physically relevant.

In general, Equation (7) presents a system of linear equations, with a superoperator acting on the unknown density matrix. If the density matrix has size N×N, the superoperator will be a matrix of size $N^{2}\times N^{2}$, and when exploiting its sparse structure of the Hamiltonian ${\hat{H}}_{R}$ and of the dissipative Lindblad terms, finding the solution of Equation (7) becomes numerically prohibitive. Building the sparse superoperator matrix can be numerically demanding in terms of memory.

Thus, we used an alternative approach, exploiting as much as possible the similarity between the Lindblad equation and Sylvester equations, which are matrix equations of the following form:

(8) $$\begin{array}{c} \hat{A}\hat{X}+\hat{X}\hat{B}=\hat{C} \end{array}$$

where $\hat{A},\hat{B},\hat{C}$ are known matrices and $\hat{X}$ is the unknown matrix to be found. For this class of matrix equations, more efficient solution algorithms are available [40]. In our case, we use the Bartels–Stewart algorithm [40], which has an $N^{3}$ computation cost, where N×N is the size of the density matrix.

The general steady-state Lindblad equation is as follows:

(9) $$\begin{array}{cc} L(\hat{\rho }) & =-i\left[\hat{H},\hat{\rho }\right]+\sum_{k}\left({\hat{L}}_{k}\hat{\rho }{\hat{L}}_{k}^{+}-\frac{1}{2}{\hat{L}}_{k}^{+}{\hat{L}}_{k}\hat{\rho }-\frac{1}{2}\hat{\rho }{\hat{L}}_{k}^{+}{\hat{L}}_{k}\right)=0 \end{array}$$

and is not of the Sylvester type (here, $\hat{H}$ is the Hamiltonian and ${\hat{L}}_{k}$ denotes dissipative operators). So, a direct application of the Bartels–Stewart algorithm is not possible. We, thus, split the Lindblad superoperator into a first term that resembles a Sylvester equation and a remainder, as follows:

(10) $$\begin{array}{cc} L(\hat{\rho }) & =-L_{0}\left(\hat{\rho }\right)+\epsilon L_{1}\left(\hat{\rho }\right) \end{array}$$

(11) $$\begin{array}{cc} L_{0}\left(\hat{\rho }\right) & =i\hat{H}\hat{\rho }+\frac{1}{2}\sum_{k}{\hat{L}}_{k}^{+}{\hat{L}}_{k}\hat{\rho }-i\hat{\rho }\hat{H}+\frac{1}{2}\hat{\rho }\sum_{k}{\hat{L}}_{k}^{+}{\hat{L}}_{k} \end{array}$$

(12) $$\begin{array}{cc} L_{1}\left(\hat{\rho }\right) & =\sum_{k}{\hat{L}}_{k}\hat{\rho }{\hat{L}}_{k}^{+} \end{array}$$

The steady-state density matrix $L(\hat{\rho })=0$ will be a fixed point of the following:

(13) $$\begin{array}{c} \hat{\rho }=L_{0}^{-1}L_{1}\hat{\rho } \end{array}$$

It is, thus, possible to iterate from an initial guess ${\hat{\rho }}_{m}$, solving a series of Sylvester equations, as follows:

(14) $$\begin{array}{c} L_{0}\rho_{m+1}=L_{1}{\hat{\rho }}_{m} \end{array}$$

We have found that, usually, a few (or at most, tens of) iterations are enough to find the fixed point with very high accuracy. The initial guess ${\hat{\rho }}_{0}$ for the iteration can be chosen from one of the two approximate methods presented in the following sections; we typically use ${\hat{\rho }}_{0}$, which is obtained from the summation of the rate equation perturbation theory. Due to the availability of a good initial ${\hat{\rho }}_{0}$ in our simulations, the number of Sylvester equation iterations required for convergence is small. Algorithms for solving generalized forms of the Sylvester equation have also been reported [41]; they can perhaps offer a faster converging iterative solution in cases where a good initial guess is not available. We note that the inverse of $L_{0}$ always exists in our system and the used approach allows us to compute it numerically in an efficient way.

For the validity of the Sylvester equations, the main assumption is that the RWA steady state is a valid description of the time-averaged state of the exact dynamics. The choice of the initial density matrix is not important for the final result, it only influences the convergence speed of the iterative method. As for any iterative numerical method, the initial guess for the interaction influences the number of iterations required for convergence, it will not influence the result, provided the solution is unique and the iteration converges (which is the case here). In practice, we use the guess provided by the perturbation theory and/or guesses by the steady-state density matrix already obtained for some neighboring parameter values (such guesses are easily available when computing a frequency dependence).

It is also possible to find the steady state by the numerical integration of time-dependent Lindblad Equations (4), (5), or (6). We use a standard odeint integration library [42] for the integration of equations of motion using a sparse matrix representation for all operators acting on the density matrix, which is represented as a dense matrix. The main integrator is an adaptive Runge–Kutta–Dorpi fifth-order stepping algorithm, although other integration schemes are also tested with similar results. In the time-dependent case, it is possible to check the validity of the RWA approximation since we have access to the full-time dependent evolution of the density matrix, including its oscillations around the RWA steady state, while integrating the stationary Equation (6) converges to the solution of Equation (7).

In principle, the two semi-analytical approaches to the solution of the RWA Lindblad equation at weak and strong dissipations are well known and have been used for a description of various systems. They are known as the rate equation (weak dissipation) and the semiclassical (strong dissipation) approach (see, e.g., the refs. in the subsection below). In this work, we use certain improvements of these approaches for our model system, which allow us to efficiently obtain the steady state of the Lindblad equation with a rather large number of components. Also, the comparison with the results obtained for the Lindblad equation allows us to determine the regions of validity of these approaches. We note that, in the validity regime of the semiclassical approach, there is no entanglement of qubits, even if they can be well synchronized.

### 2.2. Rate Equation Perturbation Theory

The above approach for steady-state computation can be numerically costly if an important number of Sylvester equation inversions is required. It is, thus, important to have a good initial guess for the density matrix to start the iteration. In the limit where the damping terms in the Lindblad dynamics are weak compared to characteristic frequencies of the Hamiltonian dynamics, it is also natural to use an approximate solution based on a formal expansion of the density matrix in powers of the dissipation operators. In such an approach, the dominant terms of the density matrix correspond to weighted eigenstates of the Hamiltonian dynamics and it can be convenient to think of this expansion as a series of rate equations describing the population of the RWA Hamiltonian eigenstates. We use this approach to obtain good initial guesses for the density matrix; it also allows us to investigate if the steady state of model Equation (6) can indeed be described by a perturbative approach based on Hamiltonian eigenstates. Heuristically, this is justified only when there is a clear separation of time scales between the Hamiltonian dynamics and the slower dissipative processes. The crossover from quantum master equations to incoherent rate dynamics is a fundamental problem that has been studied previously in different contexts [43,44,45,46]. In the following treatment, we adapt and optimize the rate equation approach for the quantum synchronization of a driven cavity coupled to qubits, thereby increasing the convergence radius of the dissipative series.

We describe this approach by starting from the general steady-state Lindblad equation and introducing a formal expansion parameter ϵ to keep track of the order of the dissipative parameters in the expansion:

(15) $$\begin{array}{cc} L(\hat{\rho }) & =-i\left[\hat{H},\hat{\rho }\right]+\epsilon \sum_{k}\left({\hat{L}}_{k}\hat{\rho }{\hat{L}}_{k}^{+}-\frac{1}{2}{\hat{L}}_{k}^{+}{\hat{L}}_{k}\hat{\rho }-\frac{1}{2}\hat{\rho }{\hat{L}}_{k}^{+}{\hat{L}}_{k}\right) \end{array}$$

(16) $$\begin{array}{cc} \; & =L_{H}\left(\hat{\rho }\right)+\epsilon L_{\gamma }\left(\hat{\rho }\right) \end{array}$$

(17) $$\begin{array}{cc} L_{H}\left(\hat{\rho }\right) & =-i[\hat{H},\hat{\rho }] \end{array}$$

(18) $$\begin{array}{cc} L_{\gamma }\left(\hat{\rho }\right) & =\sum_{k}\left({\hat{L}}_{k}\hat{\rho }{\hat{L}}_{k}^{+}-\frac{1}{2}{\hat{L}}_{k}^{+}{\hat{L}}_{k}\hat{\rho }-\frac{1}{2}\hat{\rho }{\hat{L}}_{k}^{+}{\hat{L}}_{k}\right) \end{array}$$

here, ${\hat{L}}_{k}$—as previously—denotes the relaxation operators, and $\hat{H}$ is a steady-state Hamiltonian, which in our case is given by RWA Equation (3).

To find the steady state, we need to solve $L(\hat{\rho })=0$, and we proceed to make a formal expansion in ϵ:

(19) $$\begin{array}{c} \left(L_{H}+\epsilon L_{\gamma }\right)\left({\hat{\rho }}_{0}+\epsilon {\hat{\rho }}_{1}+\ldots +\epsilon^{j}{\hat{\rho }}_{j}+\ldots \right)=0 \end{array}$$

The equations for the lowest-order approximation ${\hat{\rho }}_{0}$ are as follows:

(20) $$\begin{array}{cc} \; & L_{H}{\hat{\rho }}_{0}=0\;,\;P_{D}L_{\gamma }{\hat{\rho }}_{0}=0\;,\;\mathrm{Tr}\;{\hat{\rho }}_{0}=1 \end{array}$$

where the operator $P_{D}$ acts on a density matrix defined in the eigenbasis of $\hat{H}$ by keeping only its diagonal part. The first equation $L_{H}{\hat{\rho }}_{0}=0$ imposes that ${\hat{\rho }}_{0}$ is diagonal in the eigenbasis |n〉 of the Hamiltonian $\hat{H}=\sum_{n}\epsilon_{n}\left|n\rangle \langle n\right|$. Thus, ${\hat{\rho }}_{0}$ has the form ${\hat{\rho }}_{0}=\sum_{n}P_{n}\left|n\rangle \langle n\right|$ where $P_{n}$ can be interpreted as the probability of eigenstate |n〉. The last two equations are, thus, the rate equation determining $P_{n}$ and the normalization condition $\sum_{n}P_{n}=1$.

The solution to this equation involves building the matrix, as follows:

(21) $$\begin{array}{c} K_{nm}=\langle m|L_{\gamma }(|n\rangle \langle n|)|m\rangle . \end{array}$$

We then find non-zero solutions K|ψ〉=0, such solutions always exist since 〈1,1,…,1|K=0.

The recurrence equations for the next orders are as follows:

(22) $$\begin{array}{cc} \; & L_{H}\left(1-P_{D}\right){\hat{\rho }}_{j+1}+L_{\gamma }{\hat{\rho }}_{j}=0 \end{array}$$

which defines the off-diagonal components of ${\hat{\rho }}_{j+1}$ (in the eigenbasis of $\hat{H}$) but leaves its diagonal part undefined.

The diagonal part of ${\hat{\rho }}_{n+1}$ can be found, requiring the following:

(23) $$\begin{array}{cc} \; & P_{D}L_{\gamma }{\hat{\rho }}_{j+1}=0 \end{array}$$

this can be viewed as higher-order corrections to the rate equations changing the occupation state probabilities, $P_{n}$.

Finally, the matrix ${\hat{\rho }}_{j+1}$ is then made traceless by the transformation

(24) $$\begin{array}{c} {\hat{\rho }}_{j+1}\to {\hat{\rho }}_{j+1}-{\hat{\rho }}_{0}\mathrm{Tr}\;{\hat{\rho }}_{j+1}\;, \end{array}$$

this transformation is allowed because the induced error is of a higher order:

(25) $$\begin{array}{c} L\left({\hat{\rho }}_{0}\mathrm{Tr}\;{\hat{\rho }}_{j+1}\right)∼\epsilon^{j+2} \end{array}$$

This can also be viewed as an order-by-order normalization of the density matrix Trρ=1.

We denote as (R) the numerical result of the summation of these series. The convergence of this expansion can be checked by evaluating the residual error in Equation (19) at each step of the summation, checking that the error decreases as the number of terms grows. If the series is convergent, we continue summation until close to machine precision in the residual error, which is typically reached from the first ten terms. If the series is divergent, we stop summation when the residual error starts to increase.

We find that the direct summation of (R) seems to have a rather small convergence radius requiring the amplitude of the dissipation to be small compared to energy level splittings:

(26) $$\begin{array}{c} \gamma ,\gamma_{s}\ll \min_{i,j}\left|\epsilon_{i}-\epsilon_{j}\right| \end{array}$$

In practice, some of these transitions are almost forbidden by selection rules involving several spin flips and transitions between several oscillator quanta. We thus attempt to improve the radius of convergence of the rate equation series by incorporating dissipative terms into $L_{H}$. This is done by observing that $L_{H}$ acts on the density matrix |n〉〈m| as follows:

(27) $$\begin{array}{c} L_{H}(|n\rangle \langle m|)=i\left(\epsilon_{m}-\epsilon_{n}\right)\left|n\rangle \langle m\right| \end{array}$$

We can, thus, formally include in $L_{H}$ all the dissipative terms, which are diagonal on the same basis. This is done by introducing a new superoperator $L_{\gamma K}$, which in the eigenbasis of H is defined as follows:

(28) $$\begin{array}{c} L_{\gamma K}{\left(\hat{\rho }\right)}_{nm}=\left\{\begin{array}{cc} \left[\langle n|L_{\gamma }(|n\rangle \langle m|)|m\rangle \right]{\hat{\rho }}_{nm} & \;(m\neq n)\\ 0 & \;m=n \end{array}\right. \end{array}$$

The expansion (R) is then performed with the updated operators ${\tilde{L}}_{\gamma }$ and ${\tilde{L}}_{H}$ given by the following:

(29) $$\begin{array}{cc} {\tilde{L}}_{\gamma } & =L_{\gamma }-L_{\gamma K} \end{array}$$

(30) $$\begin{array}{cc} {\tilde{L}}_{H} & =L_{H}+L_{\gamma K} \end{array}$$

This approach has some similarities with a Jacobi preconditioning step (see, e.g., [47]), which can be used to improve the radius of convergence of iterative algorithms to solve linear equations. More physically, it corresponds to absorbing some terms of the Lindblad equation into a non-self-adjoint dissipate Hamiltonian.

We will show that this updated approach, ${\left(R\right)}^{*}$, improves the radius of convergence of the perturbation series, and for low-friction divergence, seems to occur only close to the exact resonance $\omega =\omega_{0}$, when the RWA Hamiltonian Equation (3) becomes extremely degenerate. In this case, dissipative dynamics can no longer be described satisfactorily based only on populations of the eigenstates of the Hamiltonian. Another point of view on the finite convergence radius of the rate equation series is that, formally, the series can also be summed for negative values of the dissipation rates, which then correspond to gain. The series on the dissipative and gain sides have the same radius of convergence, but if the gain is larger than some threshold, the oscillator energy will diverge. This argument provides a more physical explanation for finite convergence radius near resonance.

### 2.3. Semiclassical Approximation

While the perturbative rate equation approach works at weak dissipation, its convergence fails in the opposite limit of strong damping. In this regime, an approximate description of the system’s steady state can be obtained from a semiclassical trial density matrix. In this work, we adopt a variation of the usual semiclassical approach, where some quantum mechanical operators are replaced by their average values (for a recent example of this approach, see [48]). Instead, we apply the Lindblad superoperator to a semiclassical tensor product ansatz and minimize the residual over variational parameters, characterizing the state of the cavity and qubit polarizations. This approach provides variational approximations to the kernel of the Lindblad superoperator. The functional to be minimized can be computed analytically, and its value offers an estimation of the accuracy of the semiclassical ansatz. The possibility of checking the accuracy of the semiclassical approximation without comparing it to quantum results was one of our motivations for the variational strategy; another advantage is that since we compute the exact action of the Lindblad superoperator on the trial density matrix, some information on quantum fluctuations is preserved, which is not the case when operators are replaced by their average value.

We start by presenting this approach in the simple model case of a driven cavity, for which the RWA steady state is a pure coherent state and, thus, its minimization gives the steady state exactly and then generalizes this approach to a cavity coupled to qubits.

Our approach is to minimize the (square) matrix norm *S* of the Lindblad superoperator applied to some trial form of the density matrix, as follows:

(31) $$\begin{array}{c} S=\mathrm{Tr}\;{\left[L(\hat{\rho })\right]}^{+}L\left(\rho \right) \end{array}$$

We first consider the simple model of a driven dissipative cavity in the RWA, as follows:

(32) $$\begin{array}{cc} \hat{H} & =\omega_{r}{\hat{a}}^{+}\hat{a}+F\left(\hat{a}+{\hat{a}}^{+}\right) \end{array}$$

(33) $$\begin{array}{cc} L(\hat{\rho }) & ={\left[L(\hat{\rho })\right]}^{+}=-i\left[\hat{H},\hat{\rho }\right]+\gamma \left(\hat{a}\hat{\rho }{\hat{a}}^{+}-\frac{1}{2}{\hat{a}}^{+}\hat{a}\hat{\rho }-\frac{1}{2}\hat{\rho }{\hat{a}}^{+}\hat{a}\right) \end{array}$$

where $\omega_{r}=\omega_{0}-\omega$ is the detuning between the cavity frequency and the driving field, *F*, at frequency ω.

We consider a trial density matrix given by a pure coherent state, parameterized by a complex $\alpha =\alpha_{x}+i\alpha_{y}$, where ($\alpha_{x}$ and $\alpha_{y}$ are the real and imaginary parts of α):

(34) $$\begin{array}{c} {\hat{\rho }}_{\alpha }=\left|\alpha \rangle \langle \alpha \right| \end{array}$$

where $\hat{a}|\alpha \rangle =\alpha |\alpha \rangle$.

The functional Equation (31) can then be evaluated as follows:

(35) $$\begin{array}{c} S_{0}\left(\alpha \right)=S(|\alpha \rangle \langle \alpha |)=2\left[F^{2}+2F\alpha_{x}\omega_{r}+\left(\alpha_{x}^{2}+\alpha_{y}^{2}\right)\omega_{r}^{2}\right]+2F\alpha_{y}\gamma +\gamma^{2}\frac{\alpha_{x}^{2}+\alpha_{y}^{2}}{2} \end{array}$$

The first bracketed term comes from the Hamiltonian dynamics while the other terms include damping effects.

We can check that the minimum $S_{0}=0$ is achieved at the following:

(36) $$\begin{array}{cc} \alpha_{x} & =-\frac{4F\omega_{r}}{\gamma^{2}+4\omega_{r}^{2}}\;,\;\alpha_{y}=-\frac{2F\gamma }{\gamma^{2}+4\omega_{r}^{2}} \end{array}$$

which is the classical response function of an oscillator at frequency $\omega_{0}$ excited at frequency ω with a detuning of $\omega_{r}=\omega_{0}-\omega$ and a relaxation time of $\tau =2\gamma^{-1}$. Since $S_{0}=0$, this solution is exact.

We now generalize this approach to the case of a cavity coupled to one and then more qubits. In the case of one-qubit with the RWA Hamiltonian Equation (3), we generalize the trial form of the density matrix to a product state:

(37) $$\begin{array}{c} \hat{\rho }=\frac{1}{2}\left|\alpha \rangle \langle \alpha \right|\left(1+b_{x}{\hat{\sigma }}_{x}+b_{y}{\hat{\sigma }}_{y}+b_{z}{\hat{\sigma }}_{z}\right) \end{array}$$

which is now parameterized by three real numbers, $b_{x,y,z}$, given the Bloch sphere coordinates of the spin density matrix, in addition to the complex number α parameterizing the cavity coherent state.

Evaluating Equation (31), we find the following:

(38) $$\begin{array}{c} S=\frac{1+b_{x}^{2}+b_{y}^{2}+b_{z}^{2}}{2}S_{0}\left(\alpha_{x},\alpha_{y}\right)+\frac{\Omega_{r}^{2}\left(b_{x}^{2}+b_{y}^{2}\right)}{2}+S_{\lambda }+S_{\gamma_{s}} \end{array}$$

where the first term corresponds to the single cavity described previously. In the second term, the frequency $\Omega_{r}=\Omega -\omega$ gives the detuning between the qubit level spacing Ω and the driving frequency. Here, we omit the qubit index, where $\Omega =\Omega_{l}$, and λ denotes the qubit–cavity interaction strength. This term just describes the qubit eigenstates without qubit–cavity coupling corresponding to $b_{x,y}=0$.

The third term, $S_{\lambda }$, describes the Hamiltonian part of the qubit–cavity coupling and all terms are proportional to λ:

(39) $$\begin{array}{cc} S_{\lambda } & =2\lambda \left[Fb_{x}+\left(\alpha_{x}b_{x}-\alpha_{y}b_{y}\right)\left(\omega_{r}-b_{z}\Omega_{r}\right)\right]+\gamma \lambda \left(\alpha_{y}b_{x}+\alpha_{x}b_{y}\right)\\ \; & +\frac{\lambda^{2}}{2}\left[4b_{z}^{2}\left(\alpha_{x}^{2}+\alpha_{y}^{2}\right)+\left(4\alpha_{x}^{2}+1\right)b_{y}^{2}+8\alpha_{x}\alpha_{y}b_{x}b_{y}+\left(4\alpha_{y}^{2}+1\right)b_{x}^{2}+{\left(b_{z}+1\right)}^{2}\right] \end{array}$$

The final term $S_{\gamma_{s}}$ contains all terms, which are induced by the qubit damping, as follows:

(40) $$\begin{array}{c} S_{\gamma_{s}}=\gamma_{s}^{2}\frac{b_{x}^{2}+b_{y}^{2}+4{\left(1+b_{z}\right)}^{2}}{8}-\gamma_{s}\lambda \left(\alpha_{y}b_{x}+\alpha_{x}b_{y}\right)\left(2+b_{z}\right) \end{array}$$

where the first term is minimized by the qubit ground states $b_{x},b_{y}=0$ and $b_{z}=-1$, while the second term mixes qubit–cavity coupling and qubit relaxation.

The cavity–qubit steady state is then obtained by minimizing *S* as a function of five parameters $\alpha_{x,y},b_{x,y,z}$. For the case of two qubits, we generalize the trial form of the density matrix to the following:

(41) $$\begin{array}{c} \hat{\rho }=\frac{1}{4}\left|\alpha \rangle \langle \alpha \right|\left(1+b_{1x}{\hat{\sigma }}_{1x}+b_{1y}{\hat{\sigma }}_{1y}+b_{z}{\hat{\sigma }}_{1z}\right)\left(1+b_{2x}{\hat{\sigma }}_{2x}+b_{2y}{\hat{\sigma }}_{2y}+b_{z}{\hat{\sigma }}_{2z}\right) \end{array}$$

the expression for *S* for this trial function is given in the Appendix A.

Our trial form for the density matrix does not include any entanglement between qubits and cavities since the trial density matrix is taken as a tensor product. The cavity’s steady state is given by a coherent state and the qubits by their Bloch sphere components. We, thus, call this the semiclassical approximation to the cavity–qubit steady state. While the minimization of the semiclassical function can no longer be performed analytically in general cases, this minimization is computationally less demanding compared to full quantum calculations. It is, thus, important to identify the regimes of the Lindblad equation, where the above semi-analytical approaches can correctly reproduce its full quantum dissipative solution.

Above, we describe the main elements of semiclassical approximation. Its applications to our model are described in the following sections. The important feature of this approximation is that it allows us to understand if the steady state of the system is close to classical behavior or not. In this way, we can see how quantum is the synchronization of qubits with the driven cavity. This semi-analytical method provides a better vision of system properties in the steady state.

## 3. Numerical and Semi-Analytical Results

In the previous section, we described how the time-dependent dissipative quantum problem of a driven cavity coupled to qubits can be transformed into a stationary problem by moving to the rotating frame. Finding the steady-state density matrix of the system is then reduced to finding the kernel of the RWA Lindblad operator $L(\hat{\rho })$. We then presented several approaches to finding this steady state. An exact numerical approach is based on iterating solutions to Sylvester equations, a weak damping perturbation theory series, which can be interpreted as population dynamics of the quantum eigenstates of the system, and a semiclassical approximation which we expect to be valid at stronger damping where quantum coherence is quickly destroyed.

Here, we first present some numerical results that confirm the convergence of the system to its RWA steady state and then investigate the different regimes of this model, where our two semi-analytical approximations apply. For most parameters, we find that either one of these approximations can be used but we also identify a regime where they are not able to describe the entangled dissipative state of the system.

### RWA Validity Analysis

To probe the validity of the RWA steady state, we consider an example of Lindblad dynamics for a single non-dissipative qubit coupled to a dissipative cavity. This model was analyzed previously in [21] using the quantum trajectory method. This method showed the bistability of the quantum trajectory wave function corresponding to two-qubit orientations even for relatively large detuning between the qubit and cavity eigenfrequencies compared to their coupling strength λ. This nontrivial regime seems to be a good test case to investigate RWA validity.

**Figure 1:**
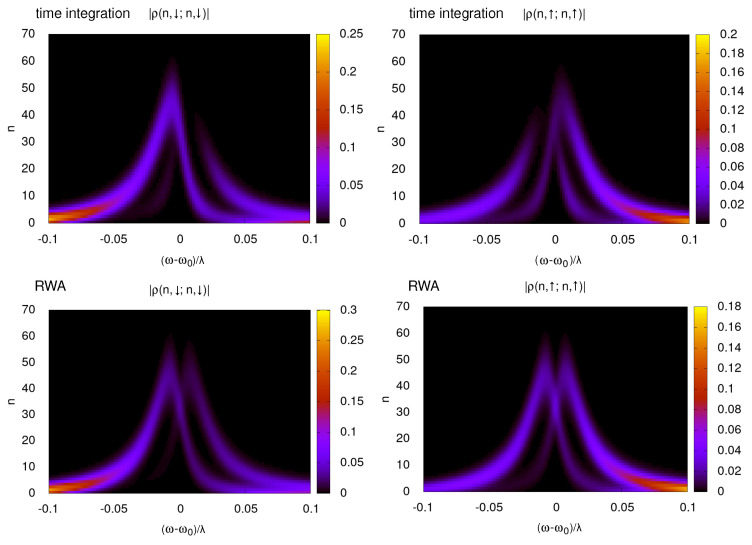
Distribution of the oscillator occupation number, *n*, as a function of the detuning from the cavity resonance $(\omega -\omega_{0})/\lambda$ and *z*-axis spin projection. The color scale shows the amplitude |ρ(n,s;n,s)| where s=↑,↓ is the spin up/down state. We remind readers that λ is the strength of the spin cavity interaction; we use it as the energy scale to facilitate the comparison between direct time integration and RWA, which depends only on the difference $\Delta =\Omega -\omega_{0}$ between the Zeeman splitting Ω and the cavity frequency $\omega_{0}$, which is set to Δ=2λ in this figure. Here, the driving strength is set to F=λ, cavity dissipation is γ=0.3λ, and qubit is dissipationless $\gamma_{s}=0$. The top rows in the figures are obtained by direct time integration for $\omega_{0}/\lambda =10$, while the bottom rows present the results from RWA. For the Lindblad time integration, numerical data show the density matrices after $\tau_{p}=2\times 10^{4}$ excitation periods, starting from the system’s ground state for F=0 at t=0 (we use the same $\tau_{p}$ for the results presentations from other cases of the time-dependent Lindblad equation). The oscillator phase spaces in the simulations were truncated to the first 100 oscillator levels (usually used for other cases).

The bistability discussed in [21] manifests as two branches of the mean cavity occupation $\langle a^{+}a\rangle$ for the two possible qubit eigenstates. This can be seen as two distinct peaks in the probability distribution of the cavity occupation number for spin-down, $P_{-}\left(n\right)=\langle n,-|\hat{\rho }|n,-\rangle$, and spin up, respectively $P_{+}\left(n\right)=\langle n,+|\hat{\rho }|n,+\rangle$, where *n* is the cavity eigenstate index. This bistability is reproduced by integrating the time-dependent Lindblad evolution in Figure 1, in the top two panels, showing $P_{-}\left(n\right)$ and $P_{+}\left(n\right)$ as functions of the detuning $\omega_{r}=\omega_{0}-\omega$ between the cavity and the driving field. Other parameters are fixed to Δ=2λ, where we introduce $\Delta =\Omega -\omega_{0}$ as the detuning between the qubit and the cavity frequencies, F=λ and γ=0.3λ. The cavity frequency is fixed to $\omega_{0}/\lambda =10$. The bottom panels in Figure 1 show the same quantities for the RWA steady state with good agreement between both methods.

While RWA qualitatively reproduces the bistability behavior, some deviations are also visible. To be more quantitative about the agreement between RWA and the exact dynamics, we consider how the mean qubit/cavity quantity dependencies on $\omega_{r}=\omega_{0}-\omega$ are changed with the increasing RWA parameter, $\omega_{0}/\lambda$, which is expected to determine the RWA validity.

**Figure 2:**
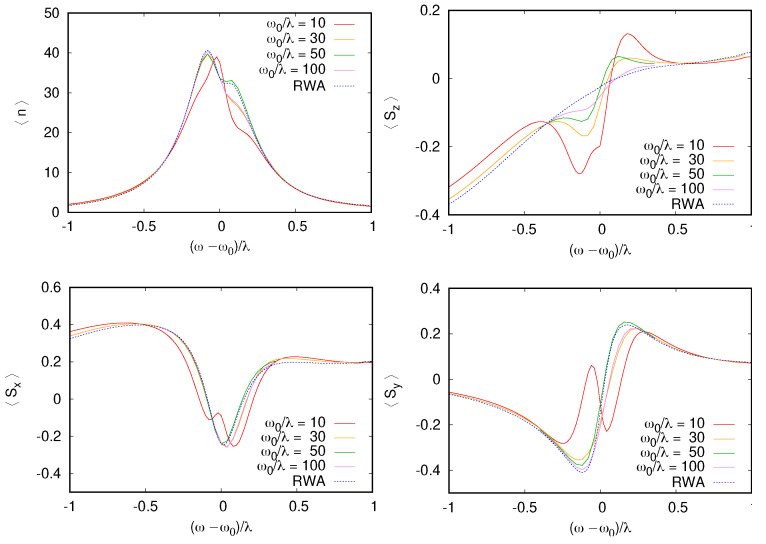
Mean spin projections $\langle S_{x,y,z}\rangle$ and oscillator quantum number $\langle n\rangle =\langle a^{+}a\rangle$ as functions of the detuning $(\omega -\omega_{0})/\lambda$ for $\Omega -\omega_{0}=2\lambda$, F=λ, γ=0.3λ (the same values as in Figure 1). Different traces correspond to quantum dynamics to increase the RWA parameter $\omega_{0}/\lambda =10,30,50,100$ (for case 50, we show a green curve on a shorter range since—outside of it all—color curves overlap, making them hardly distinguishable). The agreement with RWA improves as $\omega_{0}/\lambda$ increases but worsens a bit for the largest value. This behavior is explained as a transient effect in Figure A1 of Appendix A, where we show that relaxation to the steady state is not complete, even after $\tau_{p}=2\times 10^{4}$ microwave periods. Indeed, since the qubit is not dissipative in this simulation ($\gamma_{s}=0$), the relaxation time scale can be much longer than $\gamma^{-1}$.

**Figure A1:**
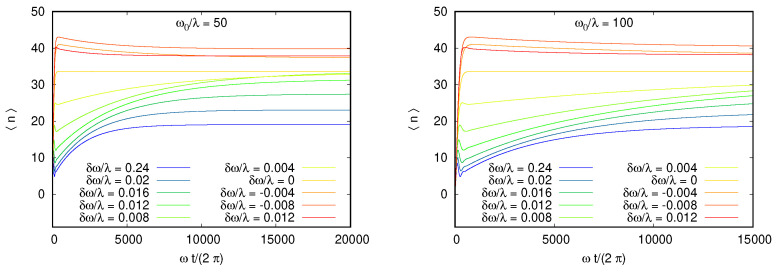
Mean oscillator quantum number $\langle n\rangle =\langle a^{+}a\rangle$ as a function of the number of driving field oscillation periods ωt/(2π) obtained by integrating the time-dependent Lindblad equation for the data in Figure 2 (we remind readers that $\Omega -\omega_{0}=2\lambda$, F=λ, γ=0.03λ). The different curves correspond to different values of $\delta \omega =\omega -\omega_{0}$. The left panel shows $\omega_{0}/\lambda =50$ and the right one 100. Relaxation is slower for positive values of δω near resonance compared to negative δω. The comparison with RWA allowed us to notice the incomplete relaxation in the simulations for $\omega_{0}/\lambda =100$, which is easy to miss because it occurs in a narrow range of δω.

The results are shown in Figure 2 for similar parameters as in Figure 1. While some deviations between the integration of the time-dependent Equation (1) and RWA are visible for ω/λ=10, the deviation rapidly drops as ω/λ increases. For $\langle {\hat{S}}_{x}\rangle$, $\langle {\hat{S}}_{z}\rangle$, and $\langle {\hat{a}}^{+}\hat{a}\rangle$, the convergence is rapid with identical results for ω/Δ=10, while the qubit projection ${\hat{S}}_{y}$ converges more slowly. In the numerical simulations, we use 100 cavity levels, which were found to be sufficient as they are twice the size of the maximum excitation number shown in Figure 1. Slow relaxation of one of the spin components in this model is shown in Figure A1 of Appendix A. RWA directly computes the steady state of the system and, thus, does not require the integration of many oscillation periods before reaching a steady state. The deviations between the RWA steady state and fully relaxed time integration are small in limit ω/λ≫1 and, thus, the RWA can be a more reliable way to explore the steady-state properties of this regime. Part of the deviations between RWA and the full-time dependent dynamics can be attributed to the slow relaxation of the spin degrees of freedom in this model; thus, integration over a long time is needed to reach the system’s steady state. Thus, overall, we find that the RWA reproduces the bistability effect quite accurately. In the quantum trajectory description, this bistability becomes visible as long-time jumps between quantum states; these jumps, on average, should reproduce the populations of each bistable state.

We highlight the very early results of the bistability of the qubit coupled to a driven resonator, as shown in [49] in the RWA approximation. However, only a specific regime, where all three frequencies of the qubit, resonator, and monochromatic drive were equal, was considered there. Also, the synchronization phenomenon was not discussed.

## 4. Weak Damping Rate Equation Approach

**Figure 3:**
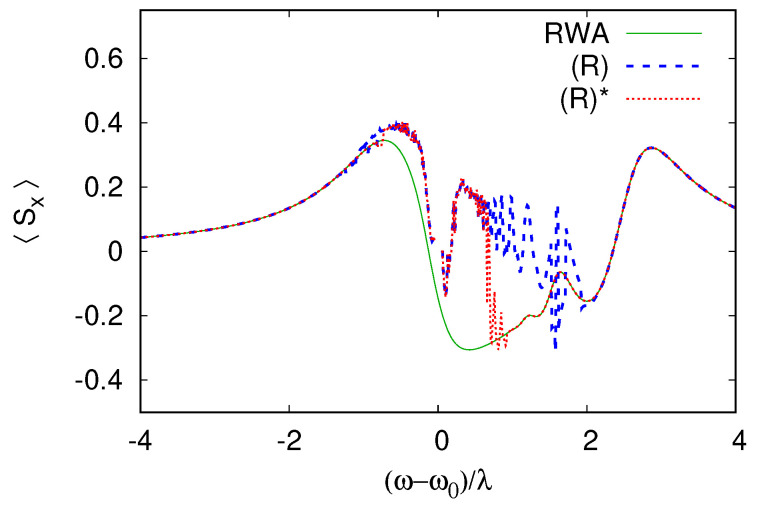
Comparison between the RWA simulation and the summation of the rate equation series for F=λ, $\Omega -\omega_{0}=2\lambda$, and $\gamma =\gamma_{s}=0.3\lambda$. The trace (R) corresponds to the summation of the series from the direct rate expansion in Equation (19), while (R)*, which exhibits a larger radius of convergence, corresponds to Equation (30). The series (R) qualitatively reproduces the position of the multiphoton resonances, but with excessive amplitude, and it fails to converge. The series (R)* reproduces multiphoton resonance accurately but still fails to converge close to the cavity resonance $(\omega -\omega_{0})/\lambda ∼1$ (further studies are needed to know if divergence occurs on energy scale λ or γ around the resonance).

We then present a typical example of the comparison between the RWA steady state and the summation of the rate equation series. We show the comparison between a single qubit interacting with a cavity in the case where both the spin and cavity are dissipative. Figure 3 shows the mean qubit spin-projection $\langle S_{x}\rangle$ in the steady state as a function of the detuning $\omega_{r}$. Similar results are obtained for other spin components $\langle S_{y,z}\rangle$. The RWA steady state found using a Sylvester equation iteration shows a series of oscillations, where $\omega_{r}$ is in the interval [0,Δ]. These oscillations correspond to multiphoton resonances in the rotating frame $k\omega_{r}=\Delta$ (where *k* is a positive integer). In the laboratory frame, these resonances correspond to transitions $\left(k+1\right)\omega =k\omega_{0}+\Omega$, where k+1 photons are absorbed to excite *k* cavity levels and the qubit at energy Ω. The direct rate equation series (R) converge only far from the resonance and fail to reproduce the multiphoton transitions but the corrected series ${\left(R\right)}^{*}$ reproduce this behavior correctly and fail to converge only in close vicinity to the cavity-driving field resonance when $\omega_{r}∼\gamma$.

**Figure 4:**
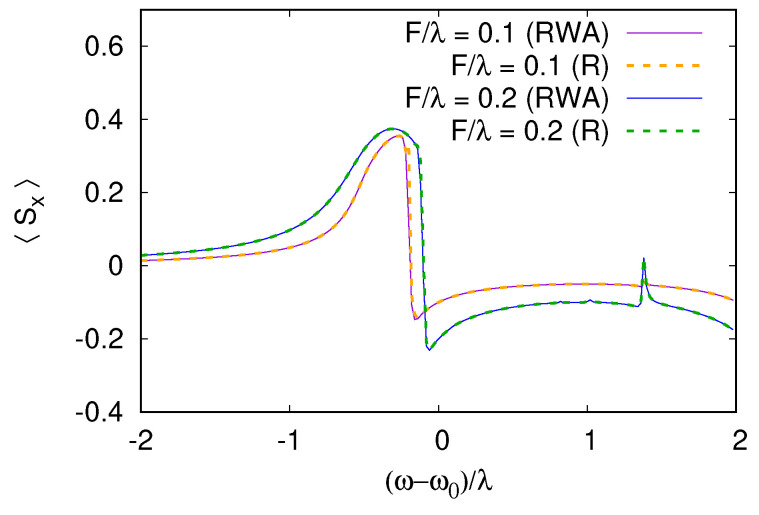
Comparison between RWA and rate equation series for weak damping $\gamma =\gamma_{s}=0.005\lambda$. As expected in this regime, the rate equation series (R), from Equation (19), converges. Excitation was reduced to avoid overheating at resonance with F=0.1λ and 0.2λ. As in the previous figures $\Omega -\omega_{0}=2\lambda$.

The comparison in Figure 3 is performed at relatively high frictions from the point of view of the rate equation perturbation theory and we see that the multi-photon resonances make the direct rate equation series (R) unstable in a wide range of $\omega_{r}$, highlighting the requirement $\gamma \ll \min_{i,j}\left|\epsilon_{i}-\epsilon_{j}\right|$. For weaker damping, the rate equation series converge in a much wider range of detuning $\omega_{r}$, as shown in Figure 4. In this case, even (R) converges almost everywhere except at very narrow resonances, where some deviation from the exact steady state is still visible.

## 5. Strong Damping Semiclassical Regime

**Figure 5:**
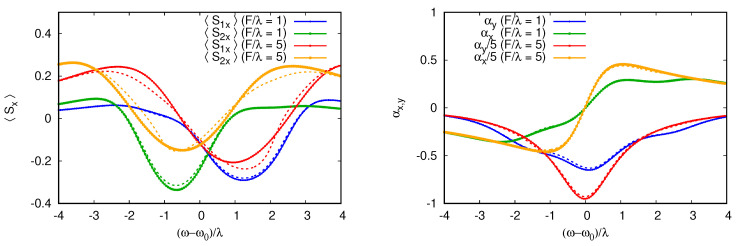
Spin projections of the two spins as functions of the detuning $(\omega -\omega_{0})/\lambda$ for excitation strength F=λ and F=5λ. Dashed lines show the spin projection predicted by the semiclassical functional Equation (38), and dotted lines show the RWA steady state. The qubit–cavity detunings are as follows: $\Delta_{1}=\Omega_{1}-\omega_{0}=2\lambda$, $\Delta_{2}=\Omega_{2}-\omega_{0}=-\lambda$. The dissipation is fixed to $\gamma =\gamma_{s}=2\lambda$, and the relatively large values of the dissipation rates ensure good agreement with the semiclassical predictions.

We find that in the opposite limit of strong damping, the semiclassical approximation is quite successful. To illustrate this, we consider a cavity coupled to two qubits with all dissipation terms in Equations (4) and (5), both for cavities and qubits with equal dissipation $\gamma =\gamma_{s}$. In this case, the dissipation is chosen to be of the same order of magnitude as the detuning between qubits and cavities. This is, thus, a regime where we expect the semiclassical theory to give a very good description of the steady state. This is confirmed by numerical simulations presented in Figure 5, which shows that both the mean spin orientation and the mean cavity susceptibility variables, αx=Re〈a^〉 and αy=Im〈a^〉, are reproduced almost perfectly by the minimization of the semiclassical functional *S*. We see that in addition to the main resonance at $\omega_{r}=0$, the cavity susceptibility shows features at resonance with the qubits $\omega =\Omega_{1,2}$, in agreement with the exact RWA solution. For these strong values of damping, the rate equation series are no longer converging for the range of detuning explored here.

**Figure 6:**
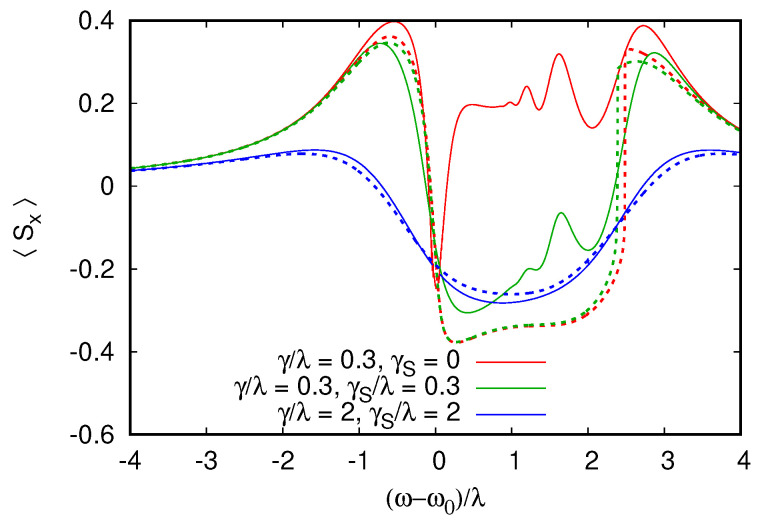
This figure compares the spin polarization $\langle S_{x}\rangle$ for one qubit coupled to a cavity for increasing dissipation rates. The polarization is shown as a function of $(\omega -\omega_{0})/\lambda$ for F=λ, $\Omega -\omega_{0}=2\lambda$. Smooth thin curves show the RWA steady state while dashed curves show the semiclassical theory. For the lowest dissipation, $\gamma_{s}=0,\gamma =0.3\lambda$, the semiclassical theory predicts a reversal of $\langle S_{x}\rangle$ in the range of $\left(\omega -\omega_{0}\right)/\lambda \in \left(0,2\right)$, which is not present in RWA, which gives the exact quantum result. But the agreement is good outside of this range. Adding some dissipation to the spin $\gamma_{s}=\gamma =0.3\lambda$ is enough to observe the polarization reversal in RWA. The agreement becomes very good for $\gamma_{s}=\gamma =2\lambda$. As expected, at a higher friction, the system behaves in a more semiclassical way.

At weaker frictions, the semiclassical results become less accurate but can still reproduce the qualitative trends of the qubit response to cavity excitation. To illustrate this, we show the semiclassical results for the case from Figure 6 for one qubit. We see that—although this method fails to capture the multiphoton resonance—the broad shape of the response is reproduced correctly.

## 6. Beyond Semiclassical Analysis and Rate Equations, Entangled Synchronization

We found that the rate equation and semiclassical approaches capture complementary regimes of the dissipative quantum dynamics with, respectively, low and high damping regimes. It is, thus, interesting if we can find a regime that is not accessible to these approaches, where an exact solution of dissipative quantum dynamics is required. This regime would have to be at a weak damping near resonance drive since this is the only regime that is outside the range of validity of both approaches. However, a strong cavity excitation at resonance can lead to an effectively semiclassical overheated regime, without special quantum coherence properties.

We, thus, consider the following setting, where two qubits are coupled to the cavity with opposite detunings from the cavity frequency $\Delta_{1}=-\Delta_{2}$ (where $\Delta_{1}=\Omega_{1}-\omega_{0}$ and $\Delta_{2}=\Omega_{2}-\omega_{0}$ are the qubit–cavity detunings). The cavity excitation at frequency $\omega =\omega_{0}$ does not break the symmetry between the qubits in RWA, and this seems to be a good regime to induce the entanglements between qubits even in the presence of damping. In the following, we analyze this model in detail with the RWA approach (6) and the exact time-dependent Lindblad dynamics (4) and (5). We also consider the validity of our two approximate approaches in this regime.

**Figure 7:**
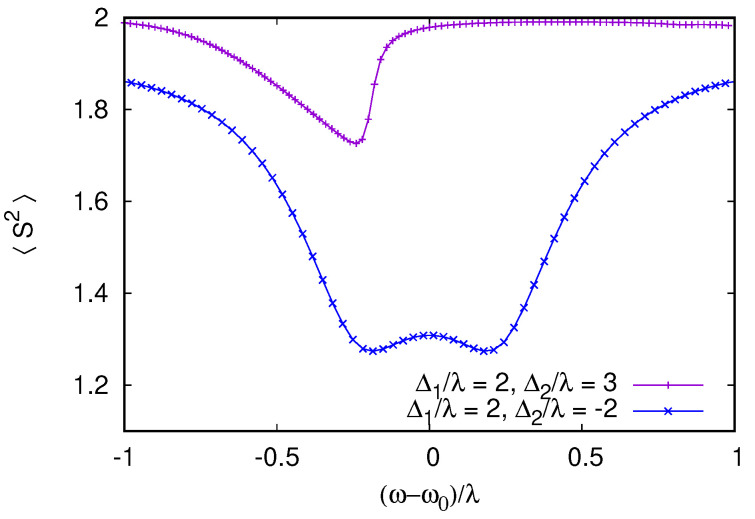
RWA calculation of the total spin of the qubit pair as a function of $(\omega -\omega_{0})/\lambda$ for two values of the qubit–cavity detuning $\Delta_{1,2}=\Omega_{1,2}-\omega_{0}$. The top curve corresponds to $\Delta_{1}/\lambda =2$ and $\Delta_{2}/\lambda =3$ with only a weak deviation from the equilibrium total spin triplet $\langle S^{2}\rangle =S\left(S+1\right)=2$. When the two Zeeman splittings are antisymmetric with respect to the cavity $\Delta_{1}=-\Delta_{2}=2\lambda$, a significantly stronger reduction of $\langle S^{2}\rangle$ is observed. Dissipative rates are set to $\gamma =\gamma_{s}=0.2\lambda$ and excitation is F=1.5λ. As previously 100 oscillator levels are used in the simulation.

To monitor the entangled state of the two detuned qubits, we consider their mean total spin operator ${\hat{S}}^{2}$${\hat{S}}^{2}$, whose expectation value is S(S+1)=2 for the triplet S=1 state of the two qubits and zero for the singlet configuration. The dependence of $\langle {\hat{S}}^{2}\rangle$ on $\omega -\omega_{0}$ is shown in Figure 7 for two cases of qubit-detuning from the cavity, comparing the antisymmetric detuning introduced above and more generic values with $\left(\Omega_{1}-\omega_{0}\right)/\lambda =2,\left(\Omega_{2}-\omega_{0}\right)/\lambda =3$. For antisymmetric detuning, a strong reduction of $\langle {\hat{S}}^{2}\rangle$ is observed when the cavity is excited at resonance; this corresponds to the dynamics of the two qubits where they rotate together, synchronizing in opposite directions and canceling (at least partially) their total spin. This reduction is not observed for the generic detuning values for which $\langle {\hat{S}}^{2}\rangle$ stays close to 2, which is its equilibrium value.

**Figure 8:**
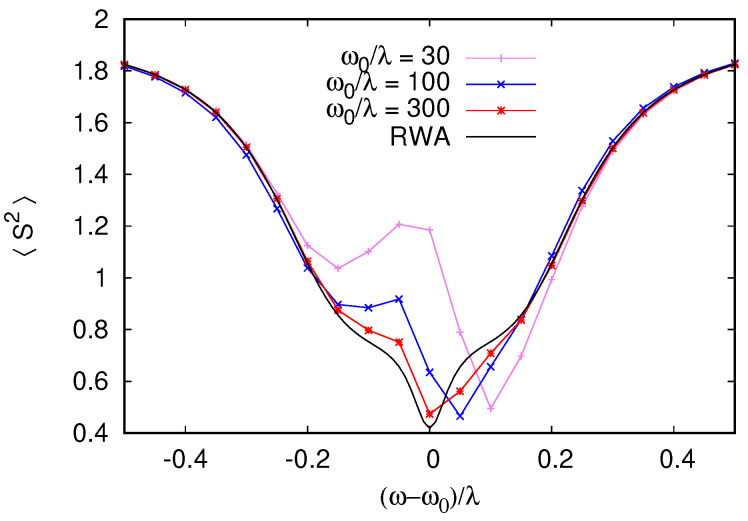
When dissipative rates are reduced compared to Figure 7, the reduction of $\langle S^{2}\rangle$ for antisymmetric qubit–cavity detunings $\Delta_{1}=-\Delta_{2}=\lambda$ becomes stronger and the singlet state of the qubit pair becomes the most probable state (75% singlet probability at the minimum of $\langle S^{2}\rangle$). Here, the driving strength is set to F/λ=0.25, the dissipative rates are γ=0.3λ with a weak qubit dissipation $\gamma_{s}=10^{-3}\gamma$ (for these parameters, at resonance, $\langle n\rangle ≃4F^{2}/\gamma^{2}≃3$). To confirm that the singlet formation is not an artifact of the RWA (black curve), we performed direct integration of the time-dependent Lindblad dynamics up to the total simulation time $3\gamma_{s}^{-1}$ for increasing RWA parameter $\omega_{0}/\lambda$ (color curves with symbols). The singlet formation is robust to non-RWA effects with the minimum $\langle S^{2}\rangle$ remaining unchanged as $\omega_{0}/\lambda$ is varied by an order of magnitude. Only weak non-RWA effects are visible as a small shift of the minimum from $\omega =\omega_{0}$ and an asymmetric $\langle S^{2}\rangle$ dependence since non-RWA effects break the symmetry between two anti-symmetrically detuned qubits.

Figure 7, corresponds to equal damping rates for cavities and qubits, which are below (but comparable to) the coupling strength γ/λ=0.2. The entanglement between the two qubits in the asymmetric detuning case can be enhanced when the damping rate of the qubits is reduced. Such a situation is shown in Figure 8 for γ/λ=0.3 and $\gamma_{s}/\lambda =3\times 10^{-3}$. The total spin $\langle {\hat{S}}^{2}\rangle$ then reduces to ≃0.5 at resonance, indicating a higher degree of anti-locking and compensation between the two qubits.

**Figure 9:**
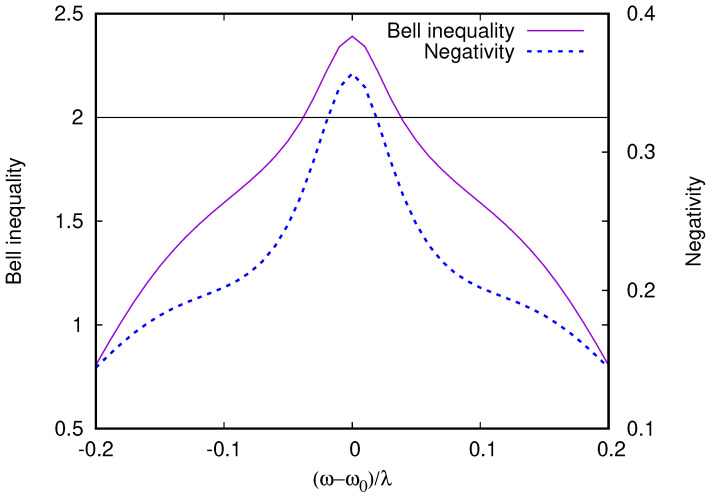
Bell inequality violation and negativity of the steady-state RWA qubit pair with the reduced density matrix (trace done over the cavity) for the parameters of Figure 8. Since the qubit pair is in a mixture of singlet and triplet states, the polarization choice for the Bell inequality has to be adjusted to observe a Bell inequality violation (see Appendix A and Figure A2). Maximal negativity for two qubits is 1/2 [50] and, thus, this steady state shows a high degree of stationary entanglement despite the dissipative decoherence of both qubits and cavities.

To confirm that this quantum synchronization and the entanglement of the two qubits is not an artifact of the RWA, we performed direct simulations of the time-dependent Lindblad dynamics of Equations (4) and (5). Figure 8 presents a comparison between RWA and dynamical Equations (4) and (5), showing almost perfect agreement for $\omega /\Delta_{1}=30$. Interestingly, even if larger deviations from RWA appear when $\omega /\Delta_{1}=30$ is lowered, the minimum $\langle {\hat{S}}^{2}\rangle$ remains constant, indicating that the anti-locking of the qubits are robust to non-RWA effects, which break the (anti)symmetry between the qubits, shifting the frequency at which the minimum $\langle {\hat{S}}^{2}\rangle$ is achieved from exact resonance. Even if the degree of cancellation between the qubit spins is not perfect, it is sufficient to generate steady states violating Bell inequalities [5]; this is shown in Figure 9. In the calculation of the Bell inequality, the spin projection has to be measured in two directions by two observers, giving the freedom to choose four possible spin projection measurement directions for the computation of the correlators. Since the entangled steady state is not a pure singlet state, we find that the violation of the Bell inequality is maximized with a ∼20° rotation of the projection directions compared to the rotation of 45°, which is used for Bell inequality tests on pure Bell (EPR) states. To avoid any dependence on the projection angles, we also show the quantum negativity (see, e.g., [50]) of the reduced qubit pair density matrix (obtained from the total density matrix by tracing over the cavity). The negativity at resonance in Figure 9 is 0.36, which indicates a substantial degree of entanglement between the two-qubit pair. We emphasize that, as opposed to entangled states generated by pulse sequences, which have finite lifetimes, this stationary entangled state is preserved in time in the presence of dissipation and decoherence within our model. The preservation of the entangled state of two qubits is also seen in numerical studies with quantum trajectory descriptions, but in those studies, the dissipation was present only for the cavity and not for qubits [22].

**Figure 10:**
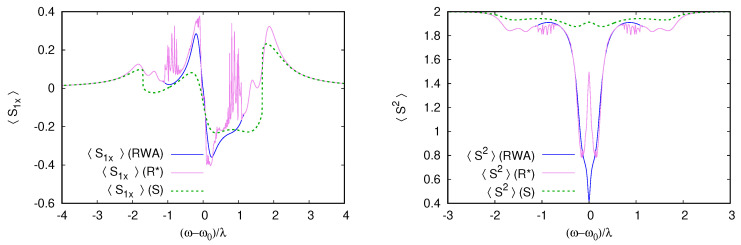
We test here if our two semi-analytic approaches can reproduce singlet formation for antisymmetric detuning presented in Figure 8 and Figure 9. The left panel shows the RWA spin projection $\langle S_{1x}\rangle$ for the first qubit compared with the rate equation series and the variational approximation, and the right panel shows the mean total spin $\langle S^{2}\rangle$. While both approaches reproduce some qualitative features, they both fail to describe the singlet formation at $\omega -\omega_{0}=0$.

To conclude this part, we investigate if this anti-locked entangled state of the qubits could be reproduced in terms of the approximate rate equation or semiclassical method. The rate equation series for $\Delta_{1}=-\Delta_{2}$ fails to converge in a rather wide range $\omega_{r}/\Delta_{1}\in \left(-1,1\right)$, probably due to resonant energy levels, which are detrimental to convergence. Treating rate equations as an asymptotic series and summing only the first terms, improving convergence, allow us to qualitatively reproduce $\langle S_{x}\rangle$ in the range of $\omega_{r}$. However, the prediction for the total spin $\langle {\hat{S}}^{2}\rangle$ is then misleading at resonance, suggesting a maximum $\langle {\hat{S}}^{2}\rangle$ in contradiction with the exact results, as illustrated in Figure 10. Not surprisingly, the semiclassical approximation only succeeds in reproducing qualitative features but completely misses the singlet formation.

In fact, the obtained singlet state represents a nontrivial example of quantum-entangled synchronization of dissipative qubits. Indeed, usually, it is expected that entanglement will disappear in a system with all elements being dissipative. However, we demonstrate that even if cavities and qubits are dissipative, we obtain the steady state where the phases of qubits are strongly correlated with each other and the phase of monochromatic driving. We discuss quantum synchronization in the following sections.

## 7. Synchronization of Several Qubits

**Figure A3:**
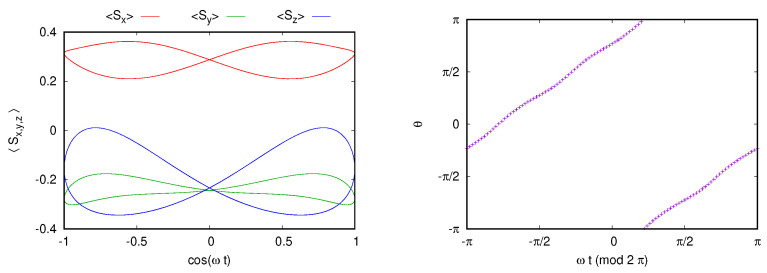
Synchronization between a driven cavity and a qubit in the system’s steady state for $\Delta_{1}=\Omega_{1}-\omega_{0}=\lambda$, $(\omega -\omega_{0})/\lambda =-0.3$, F=λ, γ=λ/3 and RWA parameter $\omega_{0}/\lambda =10$. The qubit is non-dissipative $\gamma_{s}=0$ and data are obtained by integrating Lindblad dynamics. Due to the moderate value of the RWA parameter, vibrations around the mean RWA values of the spin projections are clearly visible in the left panel. The right-hand panel shows the synchronization between the angle $\theta =\arg\langle S_{x}+iS_{y}\rangle$, which represents the in-plane spin projection of the qubit, and the phase of the cavity driving field.

In [21], using the method of quantum trajectories, it was shown that the phase of rotating qubits can be synchronized with the phase of microwave driving. We confirmed this by integrating the quantum Lindblad evolution Equations (4) and (5) (see Figure A3 of Appendix A). In fact, the quantum trajectories always produce the results with noise and it is difficult to directly see the properties of the system’s steady state. In contrast with the Lindblad description, we directly obtain the system’s steady state. The results of Figure A3 in the Appendix A directly show that in the steady state, the phase of the qubit is synchronized with the phase of monochromatic driving that corresponds to the synchronization phenomenon [2].

**Figure 11:**
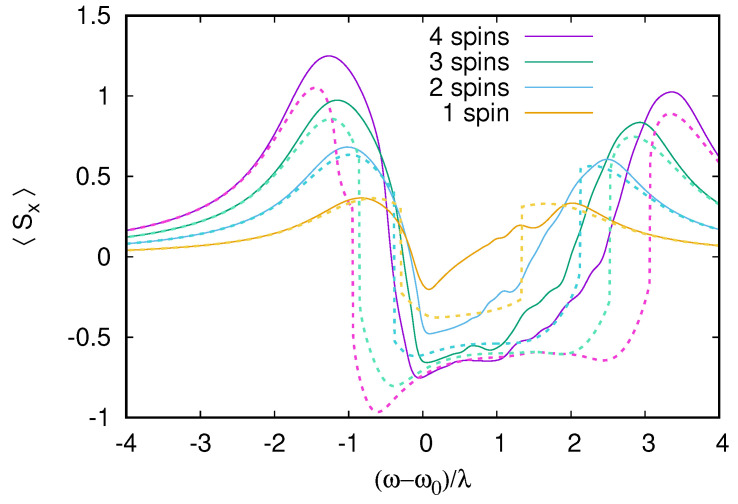
RWA calculation of total spin projection $\langle S_{x}\rangle$ of an increasing number of qubits coupled to one cavity (full curves) as a function of cavity-excitation detuning and a comparison to the semiclassical theory (dashed curves). The qubit cavity detunings are set to $\Delta_{1}=\lambda$, $\Delta_{2}=1.5\lambda$, $\Delta_{3}=2\lambda$, $\Delta_{4}=2.5\lambda$ (only the first qubits are kept when the number of qubits is smaller than four). Dissipative rates are $\gamma =\gamma_{s}=0.2\lambda$ and excitation is F=0.77λ. Even if relaxation rates are all small, the semiclassical theory still captures many properties of $\langle S_{x}\rangle$, reproducing the increase of $\langle S_{x}\rangle$ with the number of qubits, which corresponds to the synchronization of qubit rotation by the external drive. In both RWA and semiclassical data, synchronization occurs at resonance $\omega =\omega_{0}$ but perhaps less expected at a higher frequency detuned from both cavities and qubits ($(\omega -\omega_{0})/\lambda$, increasing from 2 to 3 with the number of qubits). Interestingly, the accuracy of the semiclassical approximation seems to improve with the number of qubits, which may be due to its mean-field character.

**Figure 12:**
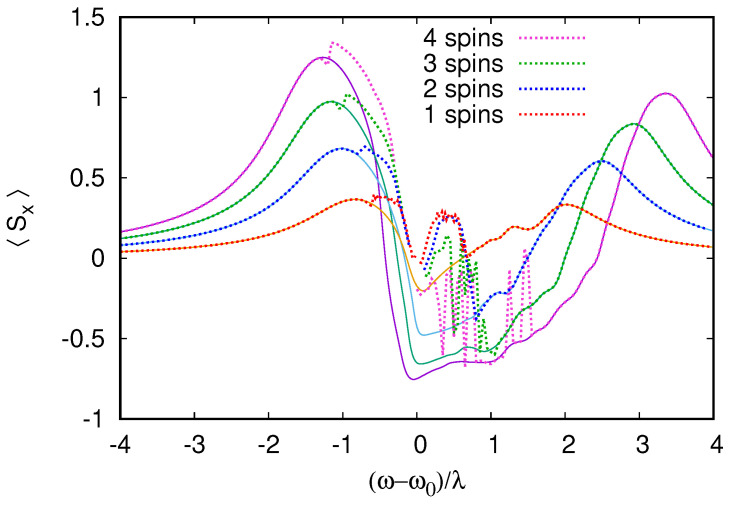
The RWA calculation of the total spin projection $\langle S_{x}\rangle$ from Figure 11 is compared to the result of the summation of the rate equation series (R)* (dotted curves). The rate equation series yield results that are nearly exact away from resonance, but they suffer from instabilities near $\omega =\omega_{0}$. It seems that the convergence range decreases with the number of qubits with various multi-qubit resonances making the series unstable even at $\omega -\omega_{0}=1.5\lambda$ for four qubits.

**Figure A4:**
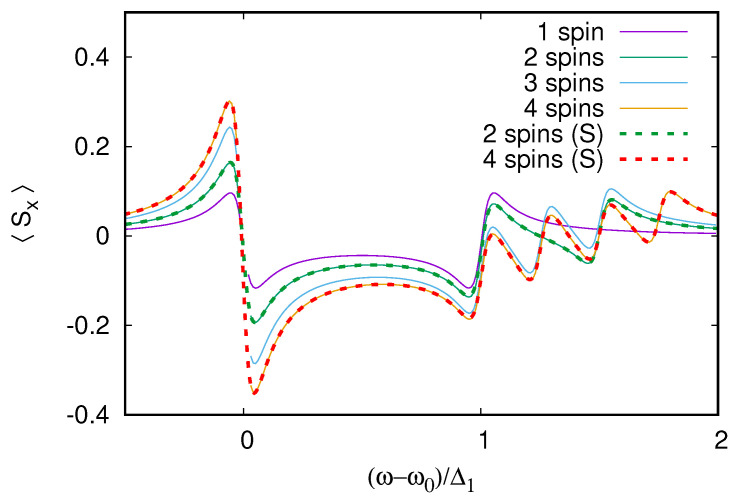
RWA calculation of the total spin projection $\langle S_{x}\rangle$ as a function of the excitation frequency-cavity detuning for an increasing number of qubits coupled to the cavity (full lines). The cavity–qubit interaction is weak λ=γ/2. The qubit–cavity detunings are set to $\Delta_{1}=10\gamma$, $\Delta_{2}=15\gamma$, $\Delta_{3}=12.5\gamma$, $\Delta_{4}=17.5\gamma$ (only the first qubits are kept when the number of qubits is smaller than four). The qubit dissipation rate is $\gamma_{s}=\gamma$ and excitation is F/γ=2.2. In this weak interaction regime, the semiclassical calculation (shown as dashed lines for 2 and 4 spins) coincides almost exactly with RWA.

**Figure A5:**
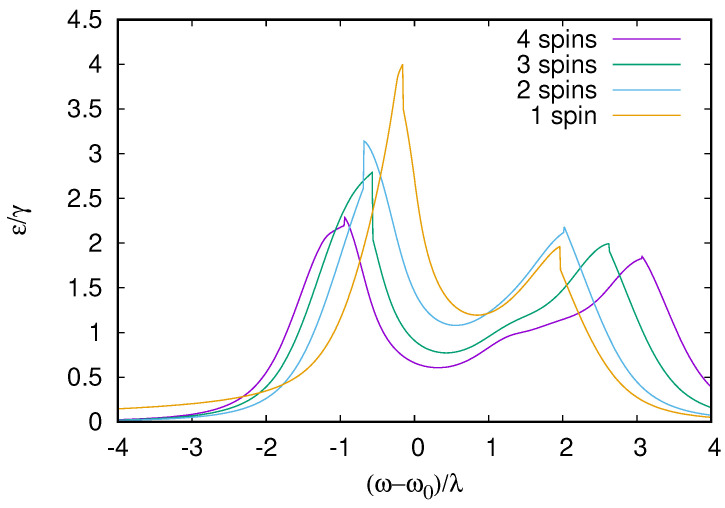
Minimum of the semiclassical functional ($\epsilon =\sqrt{S}$) rescaled by the dissipation rate $\gamma =\gamma_{s}$ for the semiclassical data from Figure 11. Good agreement between quantum and semiclassical values for the mean spin projection is observed when ϵ/γ≤1 while strong deviations are observed at peaks of ϵ/γ.

We study this synchronization effect for up to four qubits. Our results are shown in Figure 11 and Figure 12, where the RWA steady state is compared with semiclassical (Figure 11) and rate equation theories (Figure 12). All the qubits in this simulation are detuned from each other, and without external driving, their in-plane magnetization will process at different frequencies, leading to an average cancellation of the total in-plane magnetization. When the cavity is driven, two synchronization peaks appear, where the total spin projection in the rotating frame $\langle S_{x}\rangle$ grows linearly with the number of detuned qubits. The first peak is when the cavity is excited near resonance, the resonant cavity vibrations excite the qubits, which then all press at the same phase and frequency. Surprisingly, a second synchronization peak appears at a higher frequency detuned from both cavity and qubit resonances. The semiclassical approximation correctly reproduces the two synchronization peaks; however, the agreement with RWA results is only qualitative. This is because this is a weak dissipation regime with strong cavity–qubit coupling $\Delta_{1}=\lambda$ and γ/λ=0.2. In Figure A4 of Appendix A, we show that the agreement becomes almost perfect for the simpler weak coupling strong dissipation limit. We note that, in this simple regime, the linear growth of $\langle S_{x}\rangle$ with the number of qubits is observed only at cavity resonance. Figure 11 suggests that the agreement between RWA and semiclassical approximation tends to improve with the number of qubits; this may be due to the fact that our semiclassical approximation can also be viewed as a mean-field theory whose accuracy improves with more interacting qubits. The improvement in the accuracy of the semiclassical theory with a larger number of qubits (at resonance) can also be seen from the decreasing values of the semiclassical functional at its minimum (see Figure A5 of Appendix A). With this method, it is, thus, possible to obtain controlled semiclassical results for a larger number of qubits for which the exact quantum computation is no longer possible.

The summation of the rate equation series reproduces the RWA results exactly away from the cavity resonance in Figure 12; however, it seems that the diverging region around resonance grows slowly with the number of qubits.

To summarize, we find that this regime of quantum synchronization, where the total rotating in-plane spin grows with the number of qubits, is well described by both semiclassical and rate equation approaches. As discussed in the previous section, it is also possible to synchronize entangled qubits for antisymmetric qubit–cavity detunings in the vicinity of resonance between microwave driving and cavity frequency.

## 8. Discussion

In this work, we used the Lindblad equation density matrix formalism for a description of a dissipative monochromatically driven resonator (oscillator cavity) interacting with one or several qubits with a very long or moderate dissipative lifetime. This system extends the seminal Jaynes–Cummings model [35] to a dissipative regime, which is typical for superconducting qubits coupled to a driven cavity [6]. The performed numerical simulations show that the results for the full-time-dependent Lindblad equations, Equations (4) and (5), are well reproduced by the solution of the stationary RWA Lindblad equation, Equation (6). Efficient and exact methods have been developed to numerically determine the steady state under the RWA with several qubits and a driven frequency close to cavity resonance, where many cavity eigenstates are excited. For the single qubit case, a regime of qubit bistability has been established, confirming previous results obtained using the method of quantum trajectories [21].

We also developed and tested two semi-analytical approaches, which enabled us to obtain approximately accurate solutions for the exact RWA steady-state density matrix in regimes of relatively weak and strong dissipation. These correspond, respectively, to the rate equation and semiclassical approximations for the steady-state density matrix. The analytical steps of these two approaches significantly simplify the equations for the density matrix and facilitate consecutive numerical solutions, allowing us to investigate the system behavior in the vicinity of cavity resonance with many excited states. The numerical verification of these semi-analytical approaches is achieved by comparing them with the exact RWA solution, confirming their validity across a broad range of system parameters. We argue that the state of each spin is a combination of a semiclassical density matrix and quantum fluctuations. When considering the interaction of a cavity with many spins, the forces due to spin quantum fluctuations average out, and the semiclassical contribution becomes dominant. This resembles a mean-field description.

At the same time, we demonstrate the existence of system behavior that cannot be described by these semi-analytical approaches. Thus, we find that for two dissipative qubits and a dissipative driven cavity, there exists a regime when qubits remain entangled, forming a singlet, in the steady-state density matrix. The existence of such a stationary regime of entangled synchronized qubits, created by a monochromatically driven cavity, is really surprising since both qubits and cavity are dissipative. Indeed, naively one could expect that the dissipation of cavities and qubits would kill the entanglement but our results show that the entanglement may be robust and survive even in the presence of dissipation. Due to the preservation of entanglement of qubits, such a regime can be considered as a real entangled quantum synchronization.

We also identify regimes (see Figure 11) where up to four qubits are synchronized with the phase of monochromatic cavity driving, such that the total spin of the system increases proportionally to the number of qubits (spin halves) in the system. At the same time, we demonstrate that this synchronized regime is well described by the semiclassical approach. This allows us to question whether such a synchronized regime of several qubits can be considered purely quantum or rather a regime of semiclassical synchronization in the presence of strong dissipation and noise induced by quantum fluctuations. Indeed, it is known that classical synchronization is preserved in the presence of moderate noise [2]. Even if this synchronization of several qubits can be described in the frame of a semiclassical approach, and there is no entanglement, one can also argue that spin halves are purely quantum two-level systems and, hence, their synchronization is also quantum. The entangled quantum synchronization of qubits is purely quantum and cannot be obtained in the frame of the semiclassical description.

The obtained results provide a better understanding of nontrivial behavior regimes of several dissipative qubits interacting with dissipative-driven cavities. We hope that the developed methods can be useful in other contexts.

*Note:* After this work was finalized, we became aware of experiments creating two entangled qubits coupled to two resonators [51] that differed from this study, where qubits were coupled to the same resonator.

## Figures and Tables

**Figure A2:**
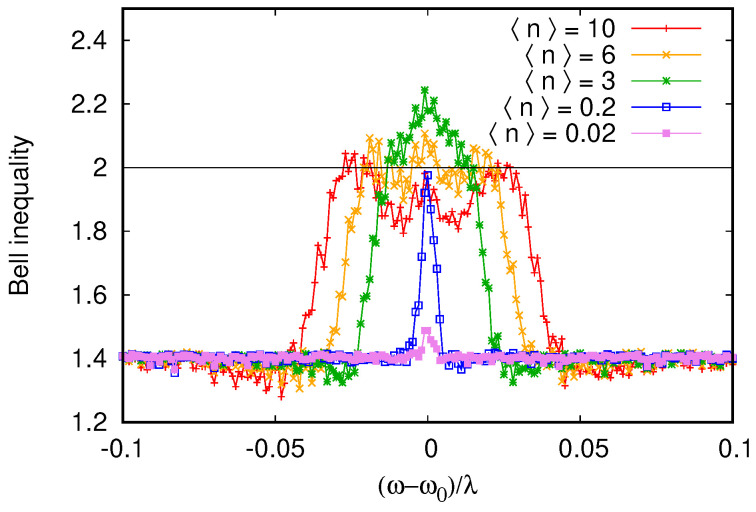
Violation of the Bell inequality for the RWA steady state for antisymmetric cavity–qubit detunings $\Delta_{1}/\lambda =-\Delta_{2}/\lambda =0.5$ (see also Figure 9). Different curves correspond to increasing driving fields, expressed as the mean cavity occupation number at resonance $\langle n\rangle =4F^{2}/\gamma^{2}$, which is shown in the legend. Dissipative rates are γ/λ=0.025 and $\gamma_{s}/\gamma =0.02$. Maximal violation of Bell inequality is observed for 〈n〉=3 (at resonance). Further increase of the driving field leads to a reduction of the Bell inequality violation, highlighting the delicate quantum nature of the singlet state. To determine the violation of Bell inequalities, a maximum was taken over randomly chosen sets of four spin projection directions for the correlation measurement.

## Data Availability

Data are contained within the article.

## Appendix A

### Appendix A.1. Semiclassical Theory for a Cavity Coupled to Several Qubits

We start by reminding readers about the RWA Hamiltonian of a qubit coupled with two cavities:

(A1) $$\begin{array}{cc} \hat{H} & =\omega_{r}{\hat{a}}^{+}\hat{a}+F\left(\hat{a}+{\hat{a}}^{+}\right)+\lambda_{1}\left(\hat{a}{{\hat{\sigma }}_{1}}^{+}+{\hat{a}}^{+}{{\hat{\sigma }}_{1}}^{-}\right)+\frac{\Omega_{r1}}{2}{\hat{\sigma }}_{1z}+\lambda_{2}\left(\hat{a}{{\hat{\sigma }}_{2}}^{+}+{\hat{a}}^{+}{{\hat{\sigma }}_{2}}^{-}\right)+\frac{\Omega_{r2}}{2}{\hat{\sigma }}_{2z} \end{array}$$

where $\omega_{r}=\omega_{0}-\omega$ and $\Omega_{r1}=\Omega_{1}-\omega ,\Omega_{r2}=\Omega_{2}-\omega$.

We analytically compute and then minimize the following:

(A2) $$\begin{array}{c} S_{2}=\mathrm{Tr}\;{\left[L(\hat{\rho })\right]}^{+}L\left(\rho \right) \end{array}$$

over trial density matrices, which are given by the semiclassical ansatz:

(A3) $$\begin{array}{c} \hat{\rho }=\frac{1}{4}\left|\alpha \rangle \langle \alpha \right|\left(1+\rho_{1x}{\hat{\sigma }}_{1x}+\rho_{1y}{\hat{\sigma }}_{1y}+\rho_{z}{\hat{\sigma }}_{1z}\right)\left(1+\rho_{2x}{\hat{\sigma }}_{2x}+\rho_{2y}{\hat{\sigma }}_{2y}+\rho_{z}{\hat{\sigma }}_{2z}\right) \end{array}$$

For the Hamiltonian part of the functional, we find the following:

(A4) $$\begin{array}{cc} u_{1} & =(1+\rho_{1x}^{2}+\rho_{1y}^{2}+\rho_{1z}^{2})/2 \end{array}$$

(A5) $$\begin{array}{cc} u_{2} & =(1+\rho_{2x}^{2}+\rho_{2y}^{2}+\rho_{2z}^{2})/2 \end{array}$$

(A6) $$\begin{array}{cc} S_{0H} & =2(F^{2}+2F\alpha_{x}\omega_{r}+\left(\alpha_{x}^{2}+\alpha_{y}^{2}\right)\omega_{r}^{2}\\ S_{\lambda H}\left(\rho_{x},\rho_{y},\rho_{z},\Omega_{r}\right) & =2\lambda \left[F\rho_{x}+\left(\alpha_{x}\rho_{x}-\alpha_{y}\rho_{y}\right)\left(\omega_{r}-\rho_{z}\Omega_{r}\right)\right] \end{array}$$

(A7) $$\begin{array}{cc} \; & +\frac{\lambda^{2}}{2}\left[4\rho_{z}^{2}\left(\alpha_{x}^{2}+\alpha_{y}^{2}\right)+\left(4\alpha_{x}^{2}+1\right)\rho_{y}^{2}+8\alpha_{x}\alpha_{y}\rho_{x}\rho_{y}+\left(4\alpha_{y}^{2}+1\right)\rho_{x}^{2}+{\left(\rho_{z}+1\right)}^{2}\right]\\ S_{2H} & =S_{0H}u_{1}u_{2}+\frac{\Omega_{r1}^{2}\left(\rho_{1x}^{2}+\rho_{1y}^{2}\right)}{2}u_{2}+\frac{\Omega_{r2}^{2}\left(\rho_{2x}^{2}+\rho_{2y}^{2}\right)}{2}u_{1} \end{array}$$

(A8) $$\begin{array}{cc} \; & +u_{2}S_{\lambda_{1}H}\left(\rho_{1x},\rho_{1y},\rho_{1z},\Omega_{r1}\right)+u_{1}S_{\lambda_{2}H}\left(\rho_{2x},\rho_{2y},\rho_{2z},\Omega_{r2}\right)+\lambda_{1}\lambda_{2}\left(\rho_{1x}\rho_{2x}+\rho_{1y}\rho_{2y}\right) \end{array}$$

To write the full functional, we need to introduce the following:

(A9) $$\begin{array}{cc} S_{0} & =2\left[F^{2}+2F\alpha_{x}\omega_{r}+\left(\alpha_{x}^{2}+\alpha_{y}^{2}\right)\omega_{r}^{2}\right]+2F\alpha_{y}\gamma +\gamma^{2}\frac{\alpha_{x}^{2}+\alpha_{y}^{2}}{2} \end{array}$$

(A10) $$\begin{array}{cc} S_{\gamma_{1S}} & =-\gamma_{1S}\lambda_{1}\left(\alpha_{y}\rho_{1x}+\alpha_{x}\rho_{1y}\right)\left(2+\rho_{1z}\right)+\gamma_{1S}^{2}\frac{\rho_{1x}^{2}+\rho_{1y}^{2}+4{\left(1+\rho_{1z}\right)}^{2}}{8} \end{array}$$

(A11) $$\begin{array}{cc} S_{\gamma_{2S}} & =-\gamma_{2S}\lambda_{2}\left(\alpha_{y}\rho_{2x}+\alpha_{x}\rho_{2y}\right)\left(2+\rho_{2z}\right)+\gamma_{2S}^{2}\frac{\rho_{2x}^{2}+\rho_{2y}^{2}+4{\left(1+\rho_{2z}\right)}^{2}}{8} \end{array}$$

The full semiclassical functional of a cavity coupled to two qubits is then given by the following:

(A12) $$\begin{array}{cc} S_{2} & =S_{0}u_{1}u_{2}+\frac{\Omega_{r1}^{2}\left(\rho_{1x}^{2}+\rho_{1y}^{2}\right)}{2}u_{2}+\frac{\Omega_{r2}^{2}\left(\rho_{2x}^{2}+\rho_{2y}^{2}\right)}{2}u_{1}\\ \; & +u_{2}S_{\lambda_{1}H}+u_{1}S_{\lambda_{2}H}+\lambda_{1}\lambda_{2}\left(\rho_{1x}\rho_{2x}+\rho_{1y}\rho_{2y}\right)+\gamma u_{2}\lambda_{1}\left(\alpha_{y}\rho_{1x}+\alpha_{x}\rho_{1y}\right)+\gamma u_{1}\lambda_{2}\left(\alpha_{y}\rho_{2x}+\alpha_{x}\rho_{2y}\right)\\ \; & +u_{2}S_{\gamma_{1S}}+u_{1}S_{\gamma_{2S}}+\frac{\gamma_{1S}\gamma_{2S}\left[\rho_{1x}^{2}+\rho_{1y}^{2}+2\rho_{z1}\left(1+\rho_{1z}\right)\right]\left[\rho_{2x}^{2}+\rho_{2y}^{2}+2\rho_{z2}\left(1+\rho_{2z}\right)\right]}{8} \end{array}$$

The functional is then minimized over cavity parameters, $\alpha_{x},\alpha_{y}$, and spin projections, $\rho_{1x},\rho_{1y},\rho_{1z},\rho_{2x},\rho_{2y},\rho_{2z}$, using the NLopt optimization library.

The generalization of this functional to more qubits is a direct generalization of the two-qubit functional, keeping track of all the terms that are generated by the possible qubit combinations; for example, the term $S_{0}u_{1}u_{2}$ becomes $S_{0}u_{1}u_{2}u_{3}u_{4}$ for four qubits, $\lambda_{1}\lambda_{2}\left(\rho_{1x}\rho_{2x}+\rho_{1y}\rho_{2y}\right)$ becomes $\lambda_{1}\lambda_{2}u_{3}u_{4}\left(\rho_{1x}\rho_{2x}+\rho_{1y}\rho_{2y}\right)$, and so forth (the variables $u_{3,4}$ are defined in analogy with Equations (A4) and (A5).

### Appendix A.2. Semiclassical Theory for a Cavity Coupled to Several Qubits

Here, we provide some additional numerical data supporting the findings presented in the main text. In Figure A1, we show the slow relaxation for some values of the detuning $\omega -\omega_{0}$ in the model where the qubit is not dissipative $\gamma_{s}=0$. In Figure A2, we investigate how the maximal violation of Bell inequalities for antisymmetric qubit–cavity detuning depends on cavity driving strength. Figure A3 shows synchronized cavity–qubit behavior and oscillations around the RWA steady state due to finite values of the RWA parameter $\omega_{0}/\lambda$. Figure A4 compares the RWA steady state with the semiclassical theory for several qubits in the regime of weak qubit–cavity interaction.

### Appendix A.3. Estimation of the Semiclassical Error from the Minimum of the Semiclassical Functional

The minimal value of the functional *S* allows us to estimate the accuracy of the semiclassical ansatz $\rho_{s}$ with respect to the exact steady state for which L(ρ)=0. Let $\rho_{s}$ be the semiclassical density matrix, realizing the minimum of $\epsilon^{2}=S{\left(\rho_{s}\right)}^{2}={\left|L\left(\rho_{s}\right)\right|}^{2}$, where |.| denotes the matrix norm ${\left|M\right|}^{2}=\mathrm{Tr}\;M^{+}M$ for a matrix *M*. We have the following inequality, $\epsilon \geq \;|\lambda_{1}\left|\right|\rho_{s,⊥}|$, where $|\lambda_{1}|$ is the smallest (in modulus) non-zero eigenvalue of the superoperator L and $\rho_{s,⊥}$ is the projection of $\rho_{s}$ on the subspace orthogonal to the exact solution ρ. We, thus, find the following inequality, $|\rho_{s,⊥}\left|\;\leq \epsilon /\right|\lambda_{1}|$, which binds the error of the semiclassical estimate, $\rho_{s}$. In our case, all dissipative rates are equal, $\gamma =\gamma_{s}$, and we can expect $|\lambda_{1}|∼\gamma^{-1}$, in which case, the ratio ϵ/γ gives an upper bound on the error $|\rho_{s,⊥}|$ (we need to keep in mind that since the density matrix is normalized to $\mathrm{Tr}\;\rho_{s}=1$, $|\rho_{s}|$ can be larger than 1). The numerical values of ϵ/γ for the semiclassical simulations in Figure 11 are shown in Figure A5. Comparing the two figures, we see that there is good agreement between semiclassical and quantum results when ϵ/γ≥1. At resonance $\omega =\omega_{0}$, the value of ϵ/γ decreases with the number of qubits (spins), which is consistent with the improving accuracy of the semiclassical spin projections shown in Figure 11. On the other hand, in regions of semiclassical bistability (corresponding to two almost degenerate semiclassical minima), the parameter ϵ/γ grows beyond unity, coinciding with the regions where the strongest discrepancies between semiclassical and quantum results occur.
